# Supplementary figures and images for: Novel competitive enzyme-linked immunosorbent assay for the detection of the high-risk Human Papillomavirus 18 E6 oncoprotein
Source: PLoS One. 2023 Aug 15;18(8):e0290088. doi: 10.1371/journal.pone.0290088 (PMC10426986; doi:10.1371/journal.pone.0290088)

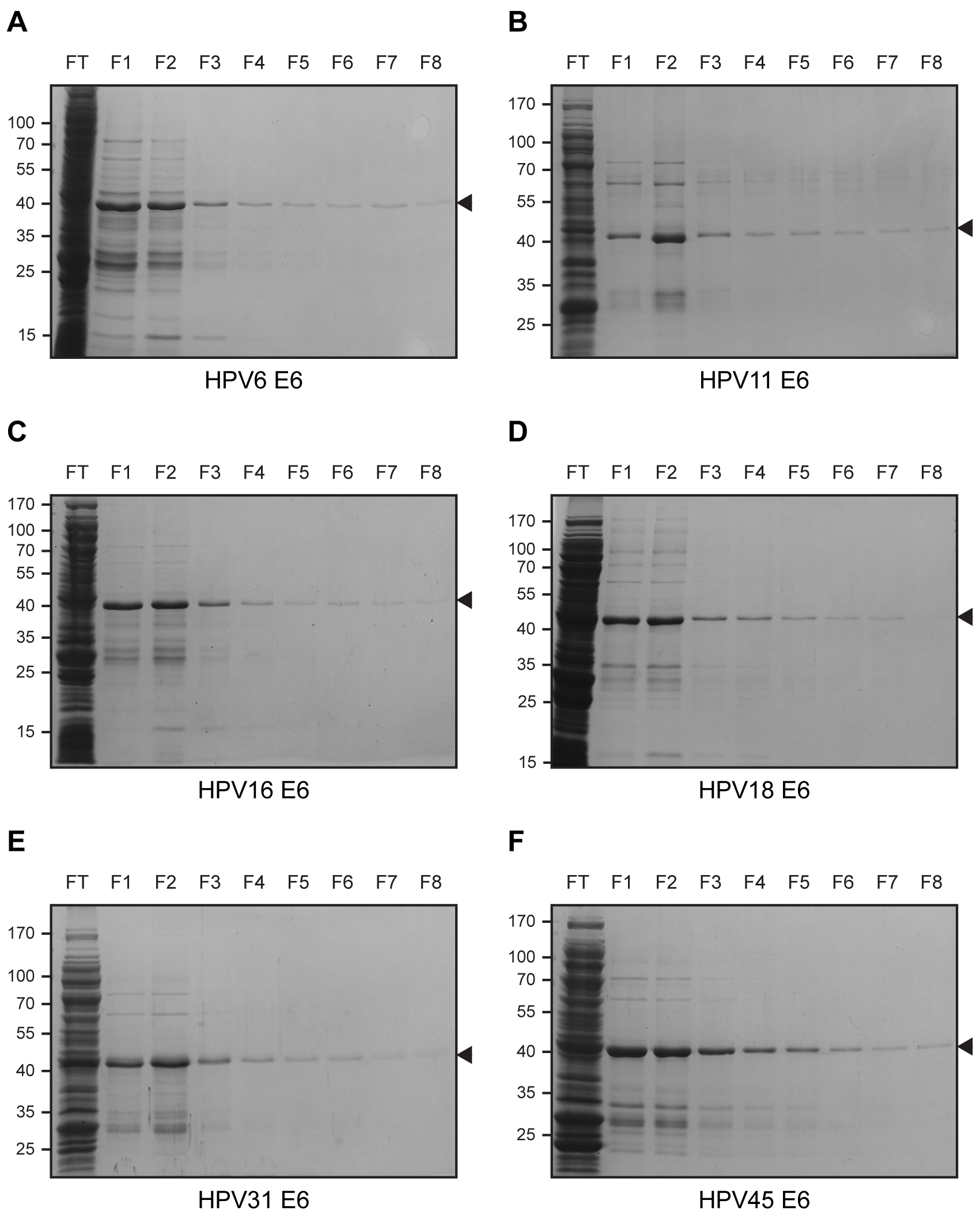

Supplement: S1 Fig — SDS-PAGE analysis of (A) HPV6, (B) HPV11, (C) HPV16, (D) HPV18, (E) HPV31 and (F) HPV45 E6 dual-tagged (GST/His) recombinant protein fractions from the IMAC purification, analyzed by Coomassie brilliant blue staining. The position of the molecular mass standards is indicated on the left. Arrowheads indicate the HPV E6 recombinant proteins. FT: flow through; F: fraction. (TIF) [file pone.0290088.s001.tif]

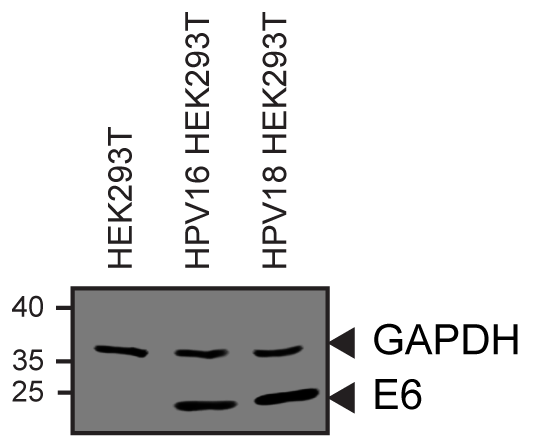

Supplement: S2 Fig — Western blot analysis of cell extracts from HPV16 E6-His and HPV18 E6-His HEK293T cells with anti-His monoclonal antibody. Untransduced HEK293T cells were used as control. GAPDH was used as loading control. The position of the molecular mass standards is indicated on the left. (TIF) [file pone.0290088.s002.tif]

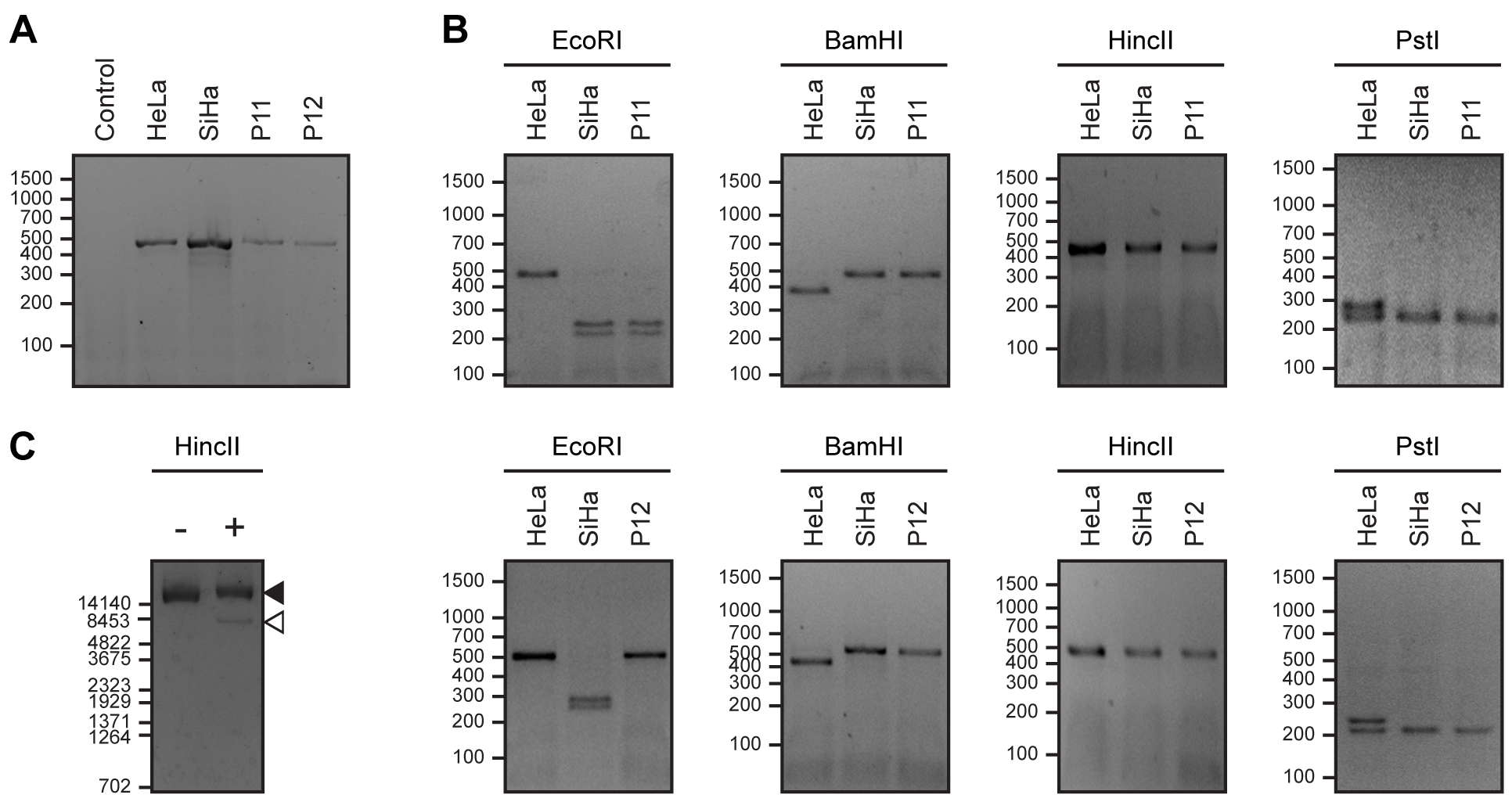

Supplement: S3 Fig — (A) Amplification by PCR with the consensus primers MY09-MY11 for the L1 gene. The negative control corresponds to molecular grade water. (B) RFLP pattern of L1 PCR product with EcoRI, BamHI, HincII and PstI. The upper pannel corresponds to patient 11 (P11) and the lower pannel corresponds to patient 12 (P12), which were characterized as HPV16 and HPV31-positive, respectively. (C) Digestion control of HincII restriction enzyme by pLB vector linearization under the same digestion conditions as L1 PCR products in (B). In (A) and (B) HeLa and SiHa cervical cancer-derived cell lines were used as HPV18 and HPV16-positive controls, respectively. In (C) the filled arrowhead shows the open circular pLB conformation, whereas the hollow arrowhead shows the linear pLB form. (TIF) [file pone.0290088.s003.tif]

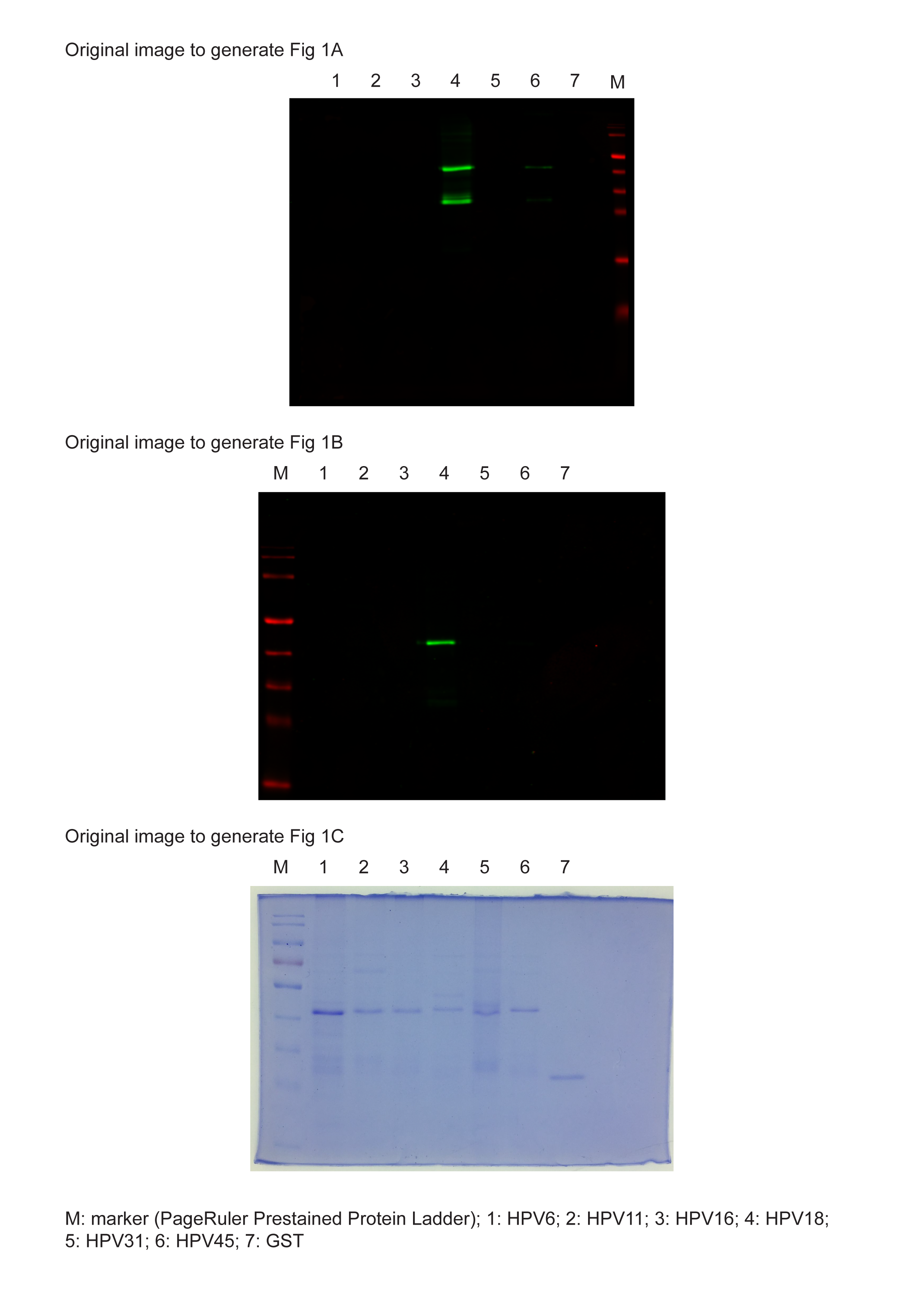

Supplement: S1 Raw images — (TIF) [file pone.0290088.s009.tif]

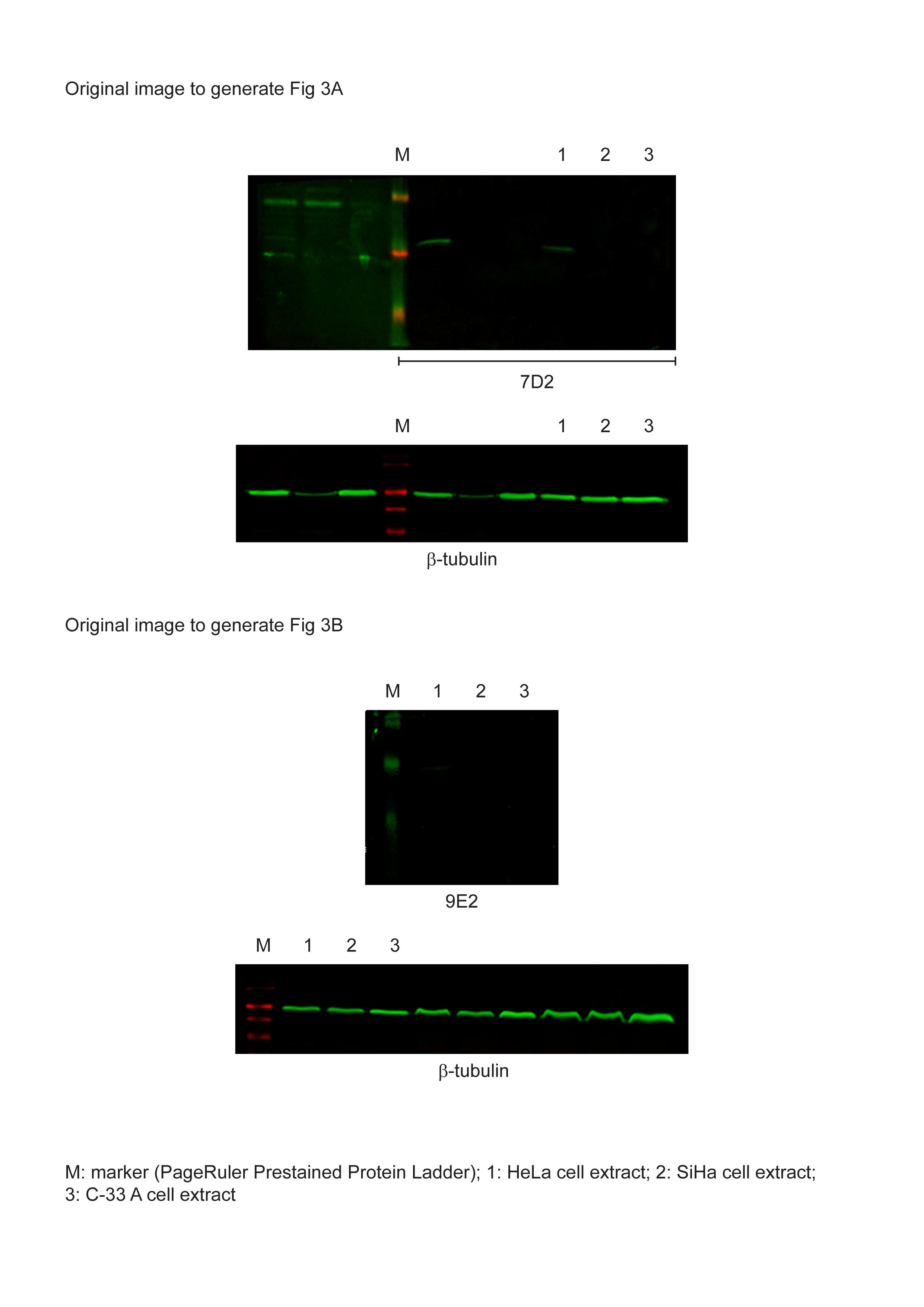

Supplement: S2 Raw images — (TIF) [file pone.0290088.s010.tif]

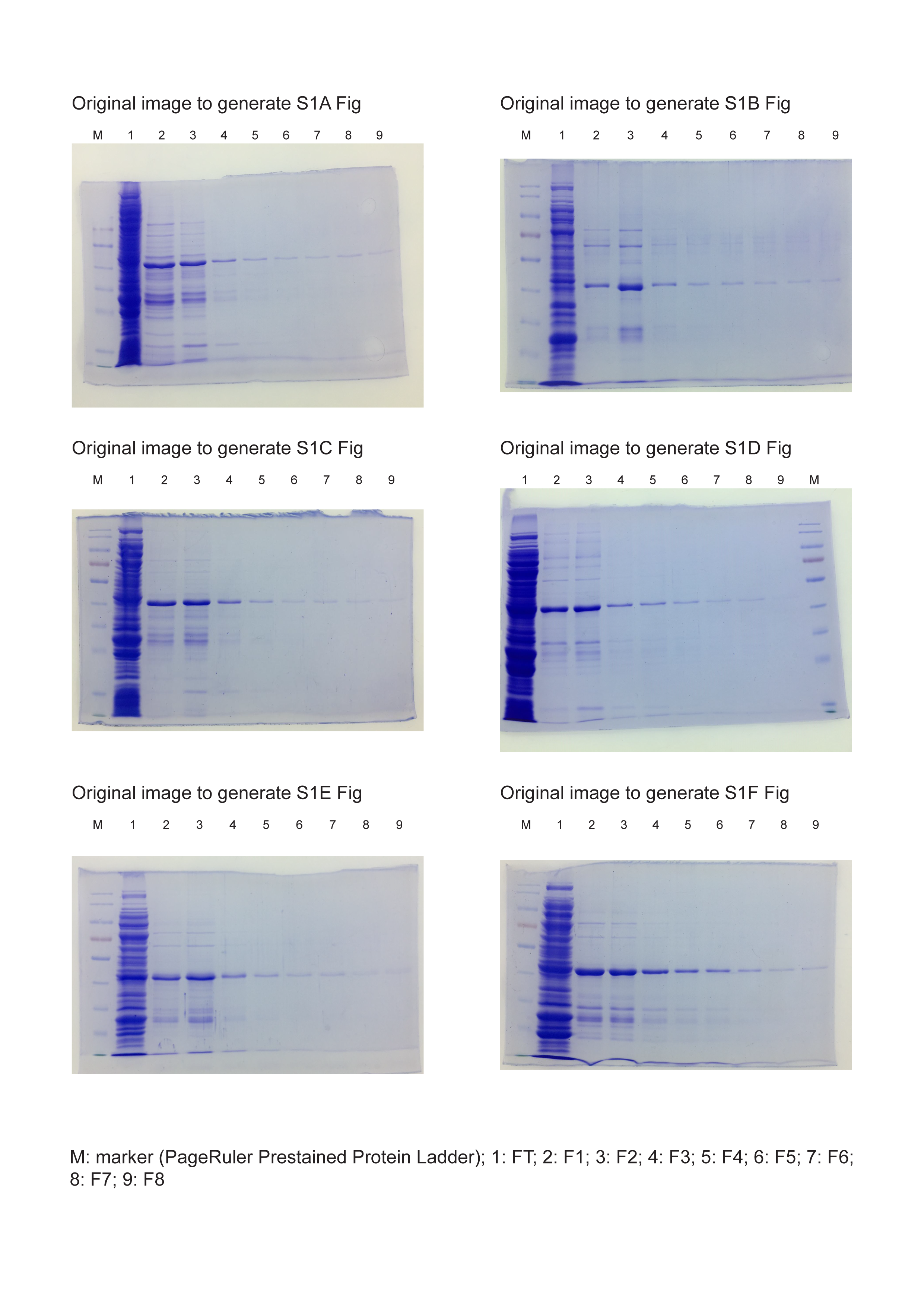

Supplement: S3 Raw images — (TIF) [file pone.0290088.s011.tif]

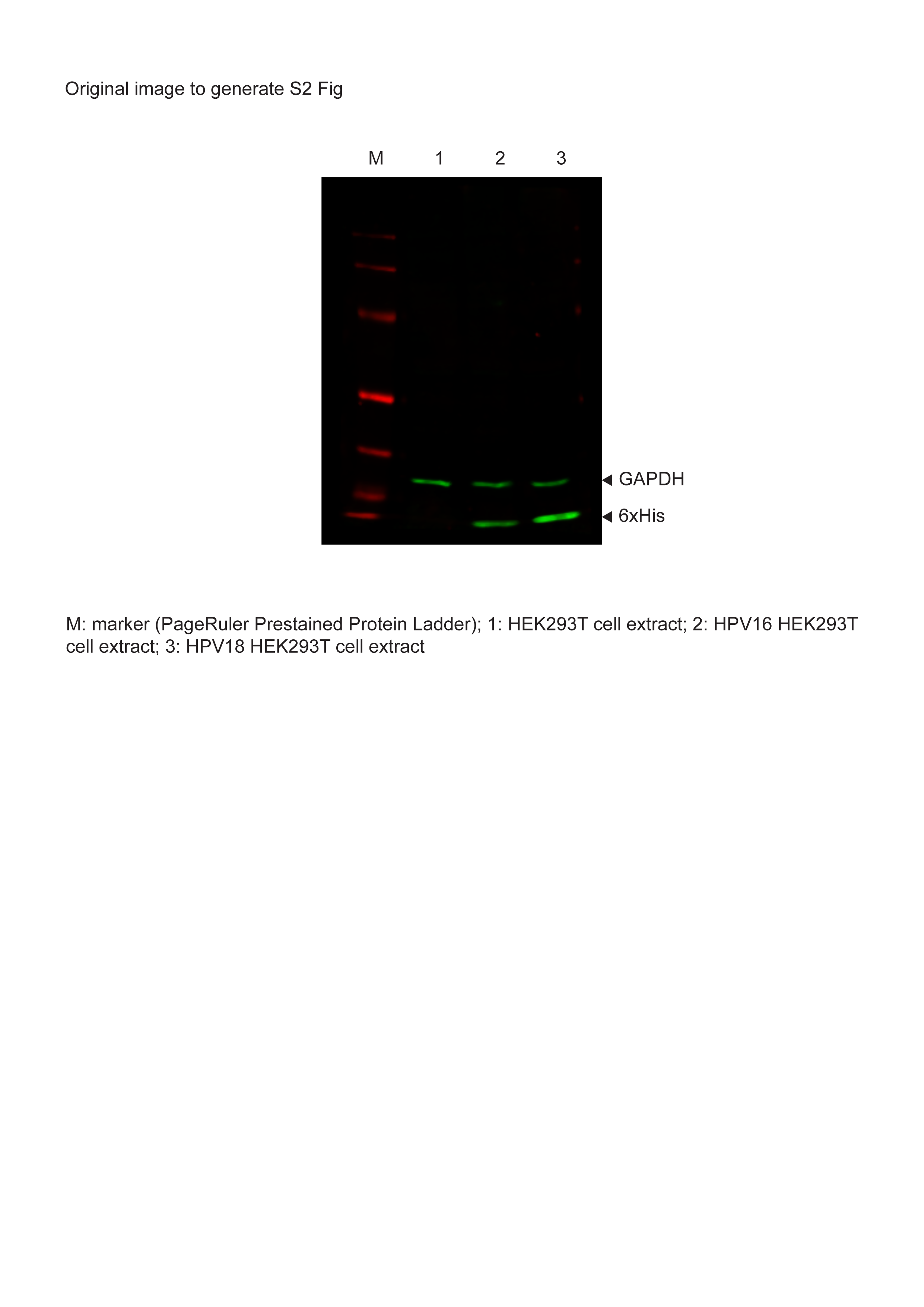

Supplement: S4 Raw images — (TIF) [file pone.0290088.s012.tif]

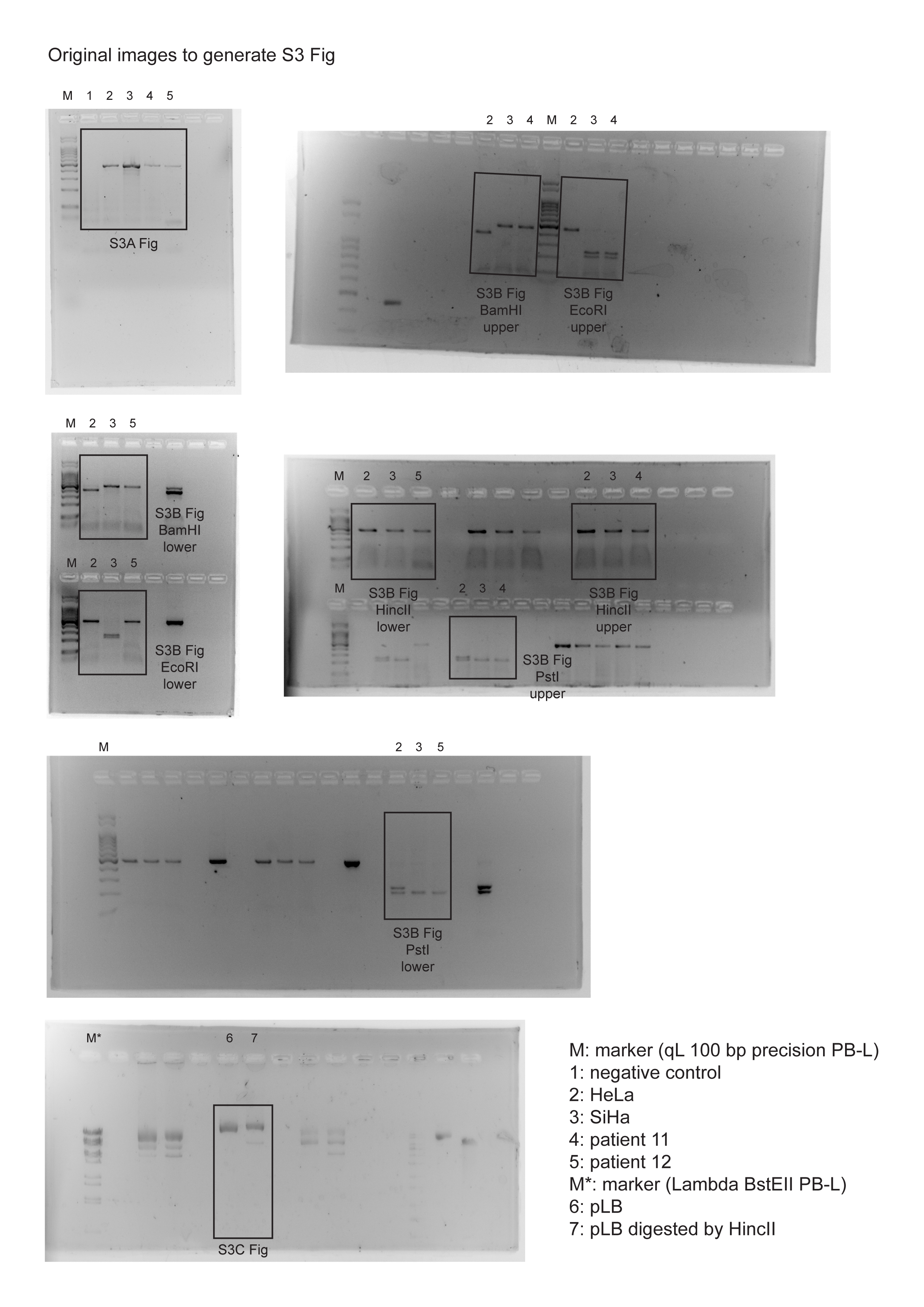

Supplement: S5 Raw images — (TIF) [file pone.0290088.s013.tif]
