# Supplementary material for: Novel competitive enzyme-linked immunosorbent assay for the detection of the high-risk Human Papillomavirus 18 E6 oncoprotein
Source: PLoS One. 2023 Aug 15;18(8):e0290088. doi: 10.1371/journal.pone.0290088 (PMC10426986; doi:10.1371/journal.pone.0290088)
Supplement: S1 Table — (DOCX) [file pone.0290088.s004.docx]

| **HPV** | **E6 UniProt Entry** |
| --- | --- |
| HPV6 | Q84291 |
| HPV11 | P04019 |
| HPV16 | P03126 |
| HPV18 | P06463 |
| HPV31 | P17386 |
| HPV45 | P21735 |
