# Supplementary material for: Novel competitive enzyme-linked immunosorbent assay for the detection of the high-risk Human Papillomavirus 18 E6 oncoprotein
Source: PLoS One. 2023 Aug 15;18(8):e0290088. doi: 10.1371/journal.pone.0290088 (PMC10426986; doi:10.1371/journal.pone.0290088)
Supplement: S2 Table — Patterns were predicted from DNA sequences of MY09-MY11 L1 PCR products. (DOCX) [file pone.0290088.s005.docx]

|  | **Uncut** | **EcoRI** | **BamHI** | **HincII** | **PstI** |
| --- | --- | --- | --- | --- | --- |
| **HPV16** | **452 bp** | 237 215 | 452 | 452 | 216 210 26 |
| **HPV18** | **455 bp** | 455 | 372 83 | 455 | 242 213 |
| **HPV31** | **452 bp** | 452 | 452 | 452 | 216 210 26 |
| **HPV33** | **449 bp** | 449 | 449 | 449 | 242 207 |
| **HPV35** | **452 bp** | 452 | 452 | 452 | 426 26 |
| **HPV39** | **455 bp** | 240 215 | 455 | 405 50 | 330 125 |
| **HPV45** | **455 bp** | 455 | 372 83 | 405 50 | 242 213 |
| **HPV51** | **452 bp** | 452 | 237 215 | 452 | 452 |
| **HPV52** | **449 bp** | 292 157 | 449 | 449 | 423 26 |
| **HPV56** | **449 bp** | 449 | 449 | 337 62 50 | 242 207 |
| **HPV58** | **449 bp** | 234 215 | 449 | 399 50 | 216 207 26 |
| **HPV59** | **452 bp** | 452 | 452 | 300 152 | 426 26 |
| **HPV68** | **455 bp** | 452 | 372 83 | 246 209 | 455 |
