# Supplementary material for: Novel competitive enzyme-linked immunosorbent assay for the detection of the high-risk Human Papillomavirus 18 E6 oncoprotein
Source: PLoS One. 2023 Aug 15;18(8):e0290088. doi: 10.1371/journal.pone.0290088 (PMC10426986; doi:10.1371/journal.pone.0290088)
Supplement: S3 Table — The concentration of HPV18 E6 of 10 different cervical cell lysates from women with normal cytology (blank samples) was calculated by the 7D2-based icELISA. (DOCX) [file pone.0290088.s006.docx]

| **Blank sample** | **Mean±SD (ng/ml)^1^** |
| --- | --- |
| Patient 1 | n.d. |
| Patient 2 | n.d. |
| Patient 3 | n.d. |
| Patient 4 | n.d. |
| Patient 5 | n.d. |
| Patient 6 | n.d. |
| Patient 7 | n.d. |
| Patient 8 | n.d. |
| Patient 9 | n.d. |
| Patient 10 | n.d. |

*^1^Data was obtained from two sample replicates*

*n.d.: not detected*
