# Supplementary material for: Novel competitive enzyme-linked immunosorbent assay for the detection of the high-risk Human Papillomavirus 18 E6 oncoprotein
Source: PLoS One. 2023 Aug 15;18(8):e0290088. doi: 10.1371/journal.pone.0290088 (PMC10426986; doi:10.1371/journal.pone.0290088)
Supplement: S4 Table — The concentration of HPV18 E6 from HPV16 and HPV31-positive clinical samples was calculated by the 7D2 icELISA. (DOCX) [file pone.0290088.s007.docx]

| **Patient** | **HR HPV type** | **Cytological report^1^** | **Mean±SD (ng/ml)^2^** |
| --- | --- | --- | --- |
| Patient 11 | HPV16 | Normal | n.d. |
| Patient 12 | HPV31 | HSIL | n.d. |

*^1^HSIL: high-grade squamous intraepithelial lesion*

*^2^Data was obtained from two sample replicates; n.d.: not detected*
