# Supplementary material for: Novel competitive enzyme-linked immunosorbent assay for the detection of the high-risk Human Papillomavirus 18 E6 oncoprotein
Source: PLoS One. 2023 Aug 15;18(8):e0290088. doi: 10.1371/journal.pone.0290088 (PMC10426986; doi:10.1371/journal.pone.0290088)
Supplement: S5 Table — (DOCX) [file pone.0290088.s008.docx]

| **HPV16**  **(UniProt P03126)** | 100 |  |  |  |
| --- | --- | --- | --- | --- |
| **HPV18**  **(UniProt P06463)** | 55.6 | 100 |  |  |
| **HPV31**  **(UniProt P17386)** | 64.4 | 51.7 | 100 |  |
| **HPV45**  **(UniProt P21735)** | 55.6 | 80.4 | 48.3 | 100 |
|  | **HPV16**  **(UniProt P03126)** | **HPV18**  **(UniProt P06463)** | **HPV31**  **(UniProt P17386)** | **HPV45**  **(UniProt P21735)** |

Sequence identity matrix showing the sequence identity on 0-100 scale among HPV16, HPV18, HPV31 and HPV45 HR HPV types. The matrix was obtained with Clustal Omega Multiple Sequence Alignment tool.
