# Supplementary material for: Novel competitive enzyme-linked immunosorbent assay for the detection of the high-risk Human Papillomavirus 18 E6 oncoprotein
Source: PLoS One. 2023 Aug 15;18(8):e0290088. doi: 10.1371/journal.pone.0290088 (PMC10426986; doi:10.1371/journal.pone.0290088)
Supplement: Attachment — Submitted filename: Review summary.pdf [file pone.0290088.s014.pdf]

In this manuscript, Contreras N.E. et al aimed at developing a icELISA to detect high risk HPV18 E6 oncoprotein. This study overall is well designed and significant to improve the diagnostic accuracy of cervical cancer. But the assay developed here can be better supported with more controls and additional experiments to be proved to be a promising novel bioanalytic method for the early detection and quantification HR HPV E6 oncoprotein. Details are listed below.

1. For the first part in the results session that identified the 7D2 mAb, it is not clear to me that if you have also identified other mAb that might be able to detect more of the HR HPV strains but not the LR strains. Is there any other antibodies that you obtain can react with all HR strain without reacting with all LR? If not, it would be more convincing if you include data from some other antibodies to that you obtain in your screening to compare with the 7D2 mAb, like the reactivity assay, wb analysis etc together with 7D2 mAb. In this way you can better prove that this 7D2 mAb is the best you get because it can at least detect the most prevalent HR HPV strain.
2. At the beginning of the second part where you claimed you established a HPV18 E6-GST concentration of 3 μg/ml fixed on the plate and a preincubation of 0.25 μg/ml 7D2 mAb with the sample for 1 h at room temperature to be the optimal assay conditions, could you please provide your data to show how the optimal concentration of E6-GST and the 7D2 mAb as well as the incubation time and temperature was determined? Not clear to me how you established and determined these variables.
3. You have used cervical cell lysates from women with negative cytology in S2 table to show your assay did not detect any signal from no cervical cancer samples, but in the whole paper you have not used any positive cancer clinical sample to test your assay, only demonstrated cell line results. I do think it is important to test in some positive clinical samples to prove your assay is capable and applicable for detecting E6 oncoprotein from clinical cervical cell lysates.

4. For the HEK293T cells data you showed in table 4, it would be better to include the HPV45 E6 expressing cells. But this is a minor point.
